# Systematic Review and Meta-Analysis of Response Rates and Diagnostic Yield of Screening for Type 2 Diabetes and Those at High Risk of Diabetes

**DOI:** 10.1371/journal.pone.0135702

**Published:** 2015-09-01

**Authors:** Kamlesh Khunti, Hamidreza Mani, Felix Achana, Nicola Cooper, Laura J. Gray, Melanie J. Davies

**Affiliations:** 1 Diabetes Research Centre, University of Leicester, Leicester General Hospital, Leicester, United Kingdom; 2 Department of Health Sciences, University of Leicester, University Road, Leicester United Kingdom; 3 Clinical Trials Unit, Medical School, University of Warwick, Coventry, United Kingdom; Hunter College, UNITED STATES

## Abstract

**Background:**

Screening for type 2 diabetes (T2DM) and individuals at risk of diabetes has been advocated, yet information on the response rate and diagnostic yield of different screening strategies are lacking.

**Methods:**

Studies (from 1998 to March/2015) were identified through Medline, Embase and the Cochrane library and included if they used oral glucose tolerance test (OGTT) and WHO-1998 diagnostic criteria for screening in a community setting. Studies were one-step strategy if participants were invited directly for OGTT and two, three/four step if participants were screened at one or more levels prior to invitation to OGTT. The response rate and diagnostic yield were pooled using Bayesian random-effect meta-analyses.

**Findings:**

47 studies (422754 participants); 29 one-step, 11 two-step and seven three/four-step were identified. Pooled response rate (95% Credible Interval) for invitation to OGTT was 65.5% (53.7, 75.6), 63.1% (44.0, 76.8), and 85.4% (76.4, 93.3) in one, two and three/four-step studies respectively. T2DM yield was 6.6% (5.3, 7.8), 13.1% (4.3, 30.9) and 27.9% (8.6, 66.3) for one, two and three/four-step strategies respectively. The number needed to invite to the OGTT to detect one case of T2DM was 15, 7.6 and 3.6 in one, two, and three/four-step strategies. In two step strategies, there was no difference between the response or yield rates whether the first step was blood test or risk-score. There was evidence of substantial heterogeneity in rates across study populations but this was not explained by the method of invitation, study location (rural versus urban) and developmental index of the country in which the study was performed.

**Conclusions:**

Irrespective of the invitation method, developmental status of the countries and or rural/urban location, using a multi-step strategy increases the initial response rate to the invitation to screening for diabetes and reduces the number needed to have the final diagnostic test (OGTT in this study) for a definite diagnosis.

## Introduction

Type 2 diabetes (T2DM) affects over 316 million people worldwide and is expected to rise to 552 million by 2030. [1] It is associated with high morbidity, mortality and healthcare costs. [2] Screening for diabetes is feasible and identifies people with modifiable cardiovascular risk. [3] Increasing global prevalence of people at high risk of diabetes [4] supports a case for early detection and initiation of treatment of T2DM to prevent a high incidence of mortality and morbidity. Screening for both diabetes and those at high risk followed by lifestyle interventions has been suggested as being cost-effective [5,6] and may reduce long term complications. [6,7] T2DM certainly meets many of the screening criteria. [8] There is evidence that screening for diabetes has an overall benefit [6] with no harm to patients. [9] Many international organisations [2,6,7,10] have produced guidelines on screening for T2DM with information on screening tests and populations, yet none have made major recommendations regarding the strategies to approach screening and the method of invitation.

There are a number of tests available for screening and diagnosing people with T2DM and those at high risk of diabetes. One of the widely used methods is the oral glucose tolerance test (OGTT).[4] However, other methods have also been suggested such as fasting plasma glucose, [7] and non-invasive risk scores such as the FINDRISC. [11] Recently glycated haemoglobin (HbA1c) has been suggested as a diagnostic tool by WHO [4] but it is still not universally standardized and many countries still recommend using OGTT. [4] NICE Guidance suggests the use of various non-invasive screening tools followed by the use of diagnostic blood tests for identifying those with diabetes or at high risk[6]. Cost, pragmatic problems in installation of a new test, and higher prevalence of conditions such as haemoglobinopathies which undermine the accuracy of HbA1c results are barriers to use this test in some developing countries.[12–15] A number of studies have reported various combinations of methods of screening including the use of risk scores which help in targeted screening followed by an OGTT. [16,17] These risk score assessments are either patient led [11] or computer generated from general practice records. [18,19]

However, there has been some uncertainty about the uptake of diabetes screening programmes in the real world setting. [20] Evidence on response to medical screening invitations has varied depending on a number of factors including the level of social deprivation[21], information on the severity of the disease, possible benefits of screening [22], the information provided [23], and levels of anxiety. [24,25] Questions still remain about how screening should be performed, in terms of producing the highest response rate and diagnostic yield.

Our objective was to perform a systematic review and meta-analysis of responses to different methods of screening and their diagnostic yield using OGTT as an example of a definite diagnostic test. We also sought to assess the effect of the countries developmental status, invitation type, locality and whether an invasive or non-invasive initial test was included on these outcomes.

## Methods

A systematic review was conducted to identify published articles on screening for T2DM. We searched Medline, Embase and Cochrane online databases (1998- March/2015) using the headings of ‘Diabetes’, ‘Pre*diabet*’, “Glycemia’, ‘Impaired glucose tolerance’, ‘Impaired fasting’, ‘Screening’, etc. (Search strategies are included in the **S1 Search Strategies**). The reference lists of all publications included at the full text review were checked manually. Only English language papers were included.

### Study selection

The initial search was performed by one reviewer and updated by another. A second reviewer reviewed all the abstracts in the first search and 30% random selection of the updated searches. All the selected full text articles were reviewed by two people. If any disagreement was not solved through discussion a third reviewer was involved. Studies were included if the subjects were free from T2DM at baseline, over the age of 18 and the screening and recruitment was population based in a community setting and the WHO 1998/9 criteria [26] was used for diagnosis and the outcome of the study included diagnostic yield.

#### Quality Assessment and Data extraction

We used established guidelines on reporting observational studies [27,28] to develop our quality assessment tool (Text and Table A in **S1 Quality Assessment**). Each reviewer scored the selected papers separately. Inter-reviewer agreement achieved in 90%.

Authors (n = 15) were contacted via e-mail for missing data and followed up after 2–3 weeks and unfortunately only five responded. Information on the study design, invitation method, risk stratification, screening steps, age, geographical location, locality of settlement (urban versus rural), were extracted. Prevalence of the diagnosis of T2DM, and those at high risk of diabetes were extracted directly and the response rate to each test was calculated using data available in the article. High risk population were defined as those with impaired glucose regulation: either impaired fasting glucose [6·1 mmol/l (110 mg/dl) ≤ fasting glucose < 7 mmol/l (140mg/dl)] or impaired glucose tolerance [7·8 mmol/l (140 mg/dl) ≤ 2 hours OGTT blood glucose < 11·1 mmol/l (200 mg/dl)] or both.

### Statistical methods

#### Outcome measures

We classified the screening strategies into one of three categories based on the number of screening steps or stages employed by individual studies. Studies were classified as a) one-step if participants were invited directly for OGTT without undergoing any pre-screening step, b) two-step if participants were invited for OGTT only after undergoing one intermediate screening assessment to identify those at high risk of diabetes and c) three/four-step if participants were invited for OGTT after undergoing pre-OGTT screening assessments at two or three intermediate stages. The type of test employed at the intermediate steps included administering *non-invasive* tools such as risk questionnaires or computer risk-scores or *invasive* techniques (blood test) such as fasting or random glucose to identify at risk individuals.

For each strategy outlined above, we considered four outcomes i) *initial response rate*; defined as the proportion of people accepted the invitation to a screening study divided by the total number invited, ii) *OGTT response rate* defined as number responding to OGTT screening invitation divided by the total number invited to OGTT, iii) *T2DM yield rate* defined as the number of newly diagnosed T2DM cases detected by OGTT divided by total number screened using OGTT and iv) *Diabetes and high risk yield rate* defined as the combined number of T2DM case and those at high risk of developing diabetes detected at the OGTT divided by total number screened using OGTT.

The response rates were applied to a hypothetical population with 1000 adults to compare the yield for each strategy (one step to multi-step) to demonstrate how the different approaches compare if these strategies were implemented in a community setting.

### Random effects model

The number of participants responding to a screening invitation or testing positive for diabetes or at risk of diabetes was assumed to be drawn from a binomial distribution so that each study independently estimated its own probability of response and a positive test. The logit of the response and yield probabilities were assumed to be drawn from a normal distribution and pooled across studies using Bayesian random effects meta-analysis.

The number needed to screen (NNS) to detect one T2DM case or the combined endpoint of “diabetes and high risk” was calculated as the inverse of the respective mean yield rates.

The association between screening outcomes (i.e. response and yield rates) and pre-specified study-level characteristics were explored and reported as response or yield rate ratios through univariate meta-regression. To calculate these rate ratios, studies were classed according to: i) the method of screening invitation or recruitment (postal/letter versus home visit/face-to-face invitation), ii) the pre-OGTT step screening tests (invasive test versus non-invasive test), iii) the developmental index of study’s country as defined by the United Nations Development programme http://hdr.undp.org/en/data (developed versus developing country), and iv) the locality of the screening (urban versus rural). The required rate ratios were defined simply as the pooled response or yield rate in one category of a study-level characteristic (for example method of invitation) divided by the corresponding pooled rate in a second category of study-level characteristic which has been designated as the reference category. For example, in the meta-regression model investigating the association between screening outcomes and method invitation, the response rate ratio was calculated as the pooled response rate in the postal invitation group divided the pooled response rate in the non-postal (home visit or face-to-face) invitation group. The yield rate ratio is defined similarly as the ratio of yield in the postal group to the yield in the non-postal group. All rate ratios were estimated on the log-odds scale to ensure the assumptions underlying the random effects meta-analysis model are applicable. Note that in the meta-regression models, studies were excluded if they provided partial or insufficient information to enable the association between a particular study-level characteristic and screening outcomes to be investigated. Heterogeneity of outcomes was quantified using the between-study standard deviation in rates on the log-odds scale.

The impact of study quality on pooled OGTT response and yield rates was investigated by restricting the analysis to studies with good quality ratings in a sensitivity analysis (Table A and B in **S1 Sensitivity Analysis**).

All analyses were carried out using Markov Chain Monte Carlo simulations implemented in the WinBUGS software version 1·4·3. [29] Minimally informative prior distributions were placed on parameters so that the analysis results are almost entirely determined by the data (model code is presented in the S1 **WinBUGS code** **: Random affects meta-analysis**). 95% credible intervals are presented throughout.

## Results

### Electronic searches and study selection

**Fig 1:**
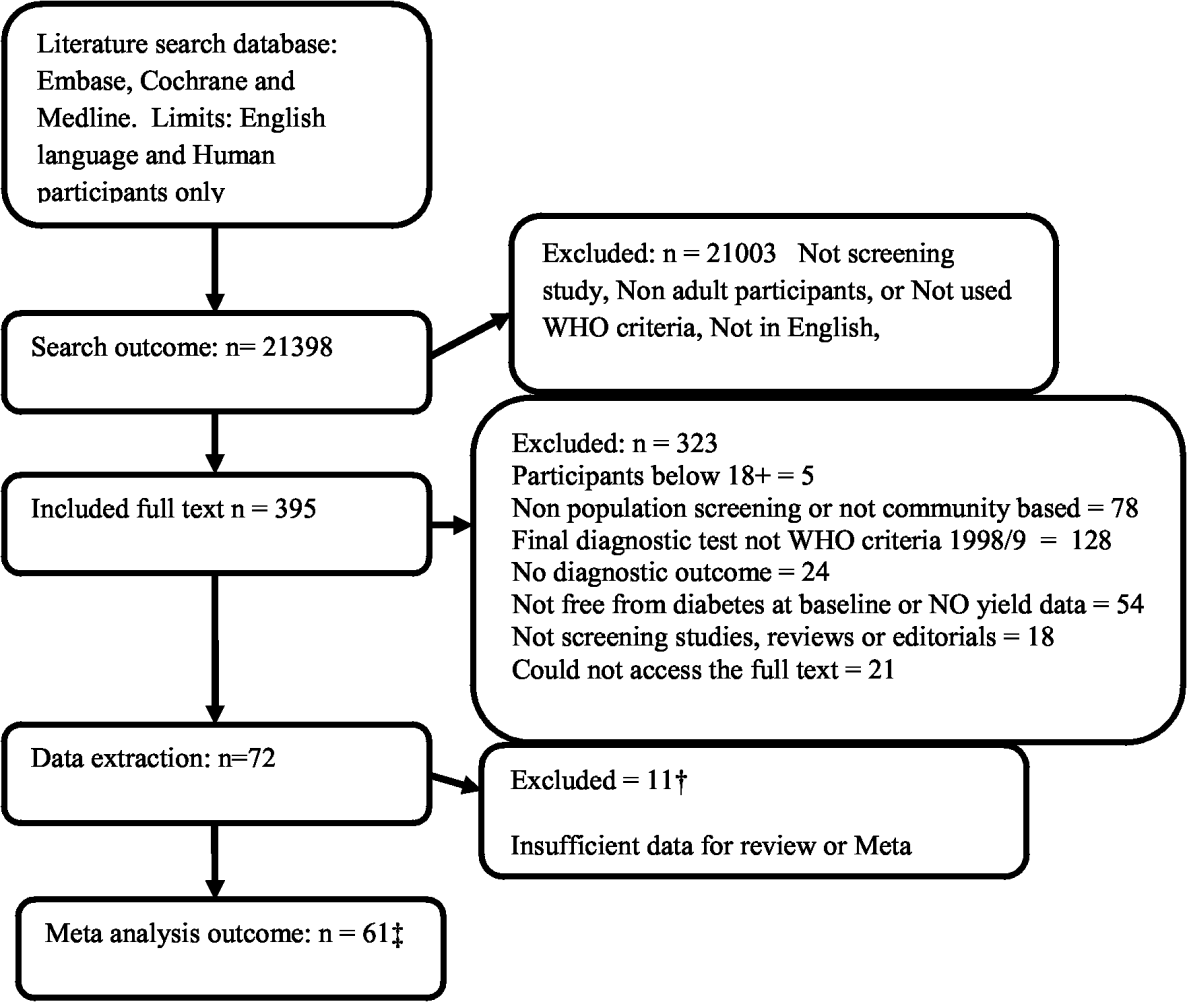
PRISMA Flowchart of paper selection. † References –30–41. ‡ This is the number of journal articles included which equals to 47 studies as some studies had reported their results in more than one paper.

The flowchart of study identification and selection has been shown in Fig 1. The search identified 21398 articles, of which 395 were selected for full text review and 72 were included for data extraction. Eleven studies [30–40] had to be excluded in this stage due to lack of data or incomplete reported data. Remaining 61 articles [41–100] were from 47 studies as some authors had published their results in multiple articles and we reviewed all these related articles to increase the accuracy of extracted data.

### Characteristic of included studies

Tables 1 and 2 present summary statistics of the remaining 47 studies included in the analysis grouped by the type of screening strategy. Twenty-three (49%) of the included studies were conducted in Europe, 16 studies (34%) in Asia, 4 (8·5%) in Australia and New Zealand, 2 (4.3%) in the United States of American and another 2 (4.3%) in Africa. The age of participants ranged from 18 to 94 years and about half of the studies were of mixed urban rural location.

**Table 1:** Characteristics of studies conducting a one- step screening strategy using only OGTT.

Lead author	Year of Publication	Country (UNDP class)†	Age group (years)	Method of invitation	Location‡	Number invited	Number screened (All steps)	Invited to OGTT step	Attended OGTT	Screened (OGTT)	T2DM (OGTT)	At risk of diabetes	Diabetes or at risk of diabetes
Riste [50]	2001	England (1)	25–79	Postal invite by GP	Urban	2018	1318	540₣	449	449	60	NA	NA
Amoah [51]	2002	Ghana (2)	25+	Home visit	Mixed	6300	4733	6300	4733	4642	209	971	1180
Botas [52]	2003	Spain (1)	30–75	Letter	Mixed	1626	1034	1626	1034	987	82	180	262
Mannuci [41]	2003	Italy (1)	30–70	Public advert	Mixed	NA	1215	NA	1215	1215	80	253	333
Rathman [53]	2003	Germany (1)	55–74	Media campaign	Mixed	2527	1653	2527	1522	1353	124	355	479
Colagirui [54]	2004	Australia (1)	25+	Home visit	Mixed	20347	15178	20293	11247	11078	416	1844	2260
Glumer [55, 56]	2004	Denmark (1)	30–60	Not stated	Mixed	12934	6906	12934	6784	6117	252	1235	1487
Habib Moeine [44]	2004	Iran (2)	30–75	Not stated	Urban	NA	NA	NA	1021	1021	60	NA	NA
Franciosi [45]	2005	Italy (1)	55–75	Recruited by GP	Mixed	NA	1840	NA	NA	1377	239	517	756
Simmons [57]	2005	Australia (1)	25+	Home visit	Rural	2372	1454	2372	1317	1317	31	149	180
Brohall [58]	2006	Sweden (1)	64	Letter	Mixed	4856	2893	4856	2595	2473	128	571	699
Heldgaard [59, 60]	2006	Denmark (1)	20–69	Not stated	Rural	2082	1374	2082	1374	1374	31	141	172
Menon [61]	2006	India (2)	18+	Home visit	Urban	3069	986	3069	986	812	85	93	178
Mohan [62–64]	2006	India (2)	20+	Home visit	Mixed	2600	2350	2600	2350	2350	222	305	527
Jia [42]	2007	China (2)	20–94	Survey	Urban	NA	NA	NA	5628	5628	276	1053	1329
Barakat [65]	2008	Oman (2)	20+	Not stated	Rural	1056	879	1056	879	830	117	167	284
El Bassuoni [49]	2008	USA (1)	18+	Community	Not stated	NA	2631	2434	1155	1155	59	231	290
Karjalainen [66]	2008	Finland (1)	45–74	Mail	Mixed	4500	2896	4500	2893	2716	236	662	898
Rush [48]	2008	New Zealand (1)	28+	Community advert	Rural	NA	5309	5309	3225	3225	161	414	575
Bener [67]	2009	Qatar (2)	20–59	Not stated	Urban	1434	1117	1434	1117	997	66	140	206
Dunkley [68]	2009	UK (1)	40–75	Letter	Mixed	11403	3241	11403	3241	3074	194	481	675
Zhou [46, 47]	2009	China (2)	21–79	Advert	Urban	NA	1107	NA	915	915	101	206	307
Gardete-Correia [69]	2010	Portugal (1)	20–79	Letter	Mixed	6161	5167	5167	4826	4826	264	1199	1463
Choi [70–73]	2011	Korea (1)	40–69	Not stated	Mixed	22772	10038	22772	9375	9375	635	NA	NA
Webb [74]	2011	UK (1)	40–75	Letter	Mixed	30950	6749	30950	6749	6749	214	1080	1294
Das [43]	2011	Bangladesh (2)	35–60	Personal contact	Urban	NA	254	NA	254	254	34	49	83
Bhansali [75]	2012	India (2)	20+	NA	Urban	2368	2245	2368	1972	1972	134	329	463
Hayes [76]	2012	UK (1)	60+	Letter	Urban	1375	584	1375	584	584	26	NA	NA
Ye [77]	2013	China	NA	Postal	Mixed	NA	NA		NA	4565	186	186	622

NA = Not reported by in published paper; OGTT = Oral glucose tolerance test

†Country classes as developed (1) and developing (2) according to United Nations Development Programme (UNDP) Human Development Index (HDI) for 2012.

‡ Mixed, refers to mixed rural/urban location ₣In this study a random sample of the initially invited people were invited to OGTT.

**Table 2:** Characteristics of studies conducting multi—step screening strategies.

Lead author	Year Published	Country (UNDP class)†	Age group (years)	Method of invitation	Location‡	Screening strategy	Number invited	Number screened (All steps)	Invited to OGTT step	Attended OGTT	Screened (OGTT)	T2DM (Any step	T2DM (OGTT)	At risk of diabetes	T2DMor at risk of diabetes
**Two steps screening strategy**															
Mcaullay [100]	2004	Australia (1)	30+	Invite by GP	Urban	HbA1c->OGTT	238	238	37	14	14	5	5	3	8
Dong [78]	2005	China (2)	20–74	household visits	Urban	FCBG->OGTT	4900	4216	469	408	408	228	228	NA	NA
Dong [78]	2005	China (2)	20–74	Household visits	Rural	FCBG->OGTT	10000	8220	786	528	528	372	372	NA	NA
Hilding [79, 80]	2006	Sweden (1)	35–56	Letter	Mixed	SQ->OGTT	12034	8108	12034	8108	7949	128	128	395	523
Korhonen [81]	2008	Finland (1)	40–75	Postal invite	Rural	SQ->OGTT	2856	2085	1756	1469	1469	65	65	375	440
Ohkuar [82]	2009	Japan (1)	35+	NA	Rural	FPG+HbA1c->OGTT	702	NA	350	220	220	20	24	85	105
Rahim [83–86]	2010	Bangladesh (2)	20+	NA	Mixed	FCBG->OGTT	5000	3981	5000	3981	3954	279	279	301	580
Makrilakis [87]	2011	Greece (1)	35–75	Recruited at GP or work place	Mixed	SQ->OGTT	6400	2000	446	222	222	50	50	NA	NA
Gray [19]	2012	UK (1)	18+	Letter	Urban	RS->OGTT	21741	4282	21741	4282	4282	180	180	1285	1465
Lin [88]	2013	China (2)	50+	Household	Rural	FBG + HbA1c->OGTT	861	453	861	453	453	54	54	96	150
Phillips [89]	2013	USA	18–87	Household	Urban	FBG->OGTT	NA	4027	1658	1581	1573	366	72	366	NA
**Three steps screening strategy**															
Spikjerman [90]	2002	Netherlands (1)	50–75	Postal invite with SRQ	Rural	RQ->FCBG->OGTT	11679	8428	532	473	473	217	181	NA	NA
Sandbaek [91, 92]	2005	Denmark (1)	40–69	Postal invite with a reminder letter	Urban	RBG/HbA1c->FPG-> OGTT	2051	1028	519	457	457	33	32	NA	NA
Janssen I [193–95]	2007	Netherlands (1)	50–70	Postal	Mixed	RQ->FPG->OGTT	27727	6855	489	397	397	397	301	NA	NA
**Four steps screening strategy**															
Christensen [92 96]	2004	Denmark (1)	40–69	Postal invite with self-assessment	Mixed	RS->RBG/HbA1c-> FBG—>OGTT	60926	30525	867	718	718	496	58	382	440
Janssen II [93–95]	2007	Netherlands (1)	50–70	Postal	Mixed	RQ->RBG->FBG-> OGTT	29251	11028	747	567	567	411	102	393	495
Sargeant [97]	2010	UK (1)	40–69	Letter	Mixed	RS->RBG->FPG-> OGTT	35539	24451	1687	1389	1389	645	645	360	1005
Park [98, 99]	2010	UK (1)	40–69	Letter	Mixed	RS->RCG->FCG-> OGTT	116	95	37	37	13	6	5	NA	NA

NA = Not reported by in published paper, Screening strategy (RS = Risk score used; RQ = Risk questionnaire–self completed; OGTT = Oral glucose tolerance test, RBG = Random blood glucose, FPG = Fasting plasma glucose).

†Country classes as developed (1) and developing (2) according to United Nations Development Programme (UNDP) Human Development Index (HDI) for 2012.

‡ Mixed Refers to mixed rural/urban location

Some studies were excluded from respective outcome analyses as they provided incomplete information on response to OGTT, [41–47] initial response, [41,42,44–50] and yield for high risk group. [41–50] Overall, 42 studies were included in the OGTT response rate, 38 in the initial response rate, and 35 in the combined “T2DM and at risk of diabetes” yield analysis.

Twenty-nine (61·7%) used OGTT alone (one-step screening approach (Table 1), 11 (23·4%) used the two-steps screening strategy, three (6·6%) used a three-step strategy and four (8·8%) used a four-step strategy. The intermediate strategies in these multi-step approaches included risk score, fasting or random glucose and HbA1c.

### Response rates

**Fig 2:**
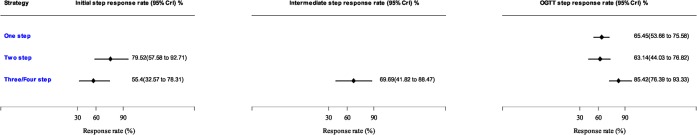
Pooled response rates to diabetes screening invitation. Estimates are from Bayesian random effects meta-analysis. CrI = Credible intervals which is similar to a confidence intervals generated using Frequentist statistics.

Estimates of pooled screening response rates are presented in Fig 2. The response rate for OGTT as a proportion of those invited for OGTT was similar in studies using one-step and two-step strategies at approximately 65% and higher in studies using a three/four-step strategy at 85·4%. Among the studies using a multi-step screening strategy, the response rate to the initial screening invitation was 79·5% for the two-step strategy which is higher than the estimated 55·4% response the rate for studies using three/four-step strategy but there was considerable overlap in the uncertainty (i.e. 95% credible intervals) around these estimates (Fig 1). Only the studies using a three/four-step strategy had an intermediate screening step which had a pooled response rate of 69·7%.

### Positive outcome at initial and intermediate screening stages

**Fig 3:**
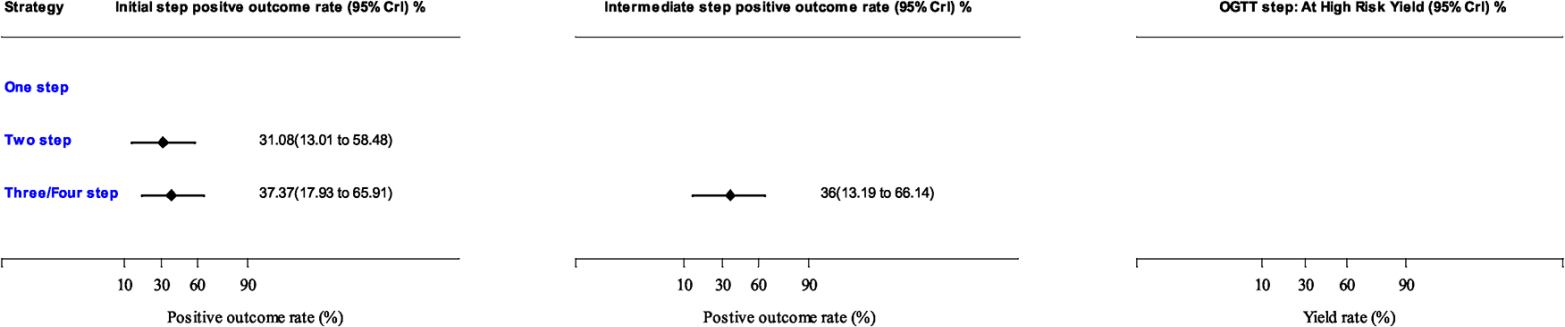
Pooled positive outcome rates of diabetes screening using risk score, risk questionnaire and impaired glucose test. Estimates are from Bayesian random effects meta-analysis. CrI = Credible intervals which is similar to confidence intervals generated using Frequentist statistics.

The proportion of individuals screened positive (i.e. identified as being at risk of T2DM) at the initial and intermediate stages of the multi-step strategies are summarised in Fig 3. These are the individuals who needed to have the next step of the screening study. The positive outcome rate was similar for two-step and three/four-step at 31.1% and 37.4% respectively at the initial step and remained relatively the same for the intermediate screening step.

### Yield rates

**Fig 4:**
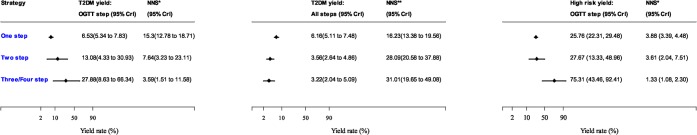
Pooled yield rates from diabetes screening meta-analysis. Estimates are from Bayesian random effects meta-analysis. CrI = Credible intervals which is similar to a confidence intervals generated using Frequentist statistics. NNS* = Number needed to screen at OGTT step to detect one case of type 2 diabetes. NNS** = Number needed to screen at initial screening step (if a multi-step strategy is used) to detect to detect one case of type 2 diabetes.

Pooled estimates of the yield for T2DM and the high risk (combined endpoint of T2DM and those at high risk of diabetes) are presented in Fig 4. Yield for OGTT T2DM increased from 6·5% (5·3, 7·8) in the one-step to 13·1% (4·3, 30·9) in the two-step and 27·9% (8·6, 66·3) in the three/four-step strategy. These yield rates correspond to number needed to screen with OGTT to detect one case of diabetes of about 15, 7.6 and about 3·5 persons respectively.

The overall T2DM yield (i.e. the total number of diabetes cases detected at all the steps as a proportion of those screened at the initial step) in the multi-step strategy studies decreased from the 6·5% in the one-step strategy reported above to 3.6% (2·6, 4.9) in the two-step strategy and 3·2% (95% CrI 2·0, to 5·1) in the three/four-step strategy. These rates corresponds to an increase in the number needed to screen at the initial step (OGTT if one-step strategy) from 15 in a one-step strategy to 28 and 31 in two-step and three/four-step strategies respectively.

Pooled estimates of the yield for the high risk (the combined endpoint of T2DM and those at high risk of diabetes) were 25·8% (22·3, 29·5), 27.7% (13·3, 49·0) and 75·3% (43·5, 92·4) for studies adopting one, two or three/four-step strategies respectively. The number needed to screen to detect one additional person with diabetes or at risk of diabetes was decreased from 4 in a one-step strategy to 3·5 and 1 in two-step and three/four-step strategies.

**Fig 5:**
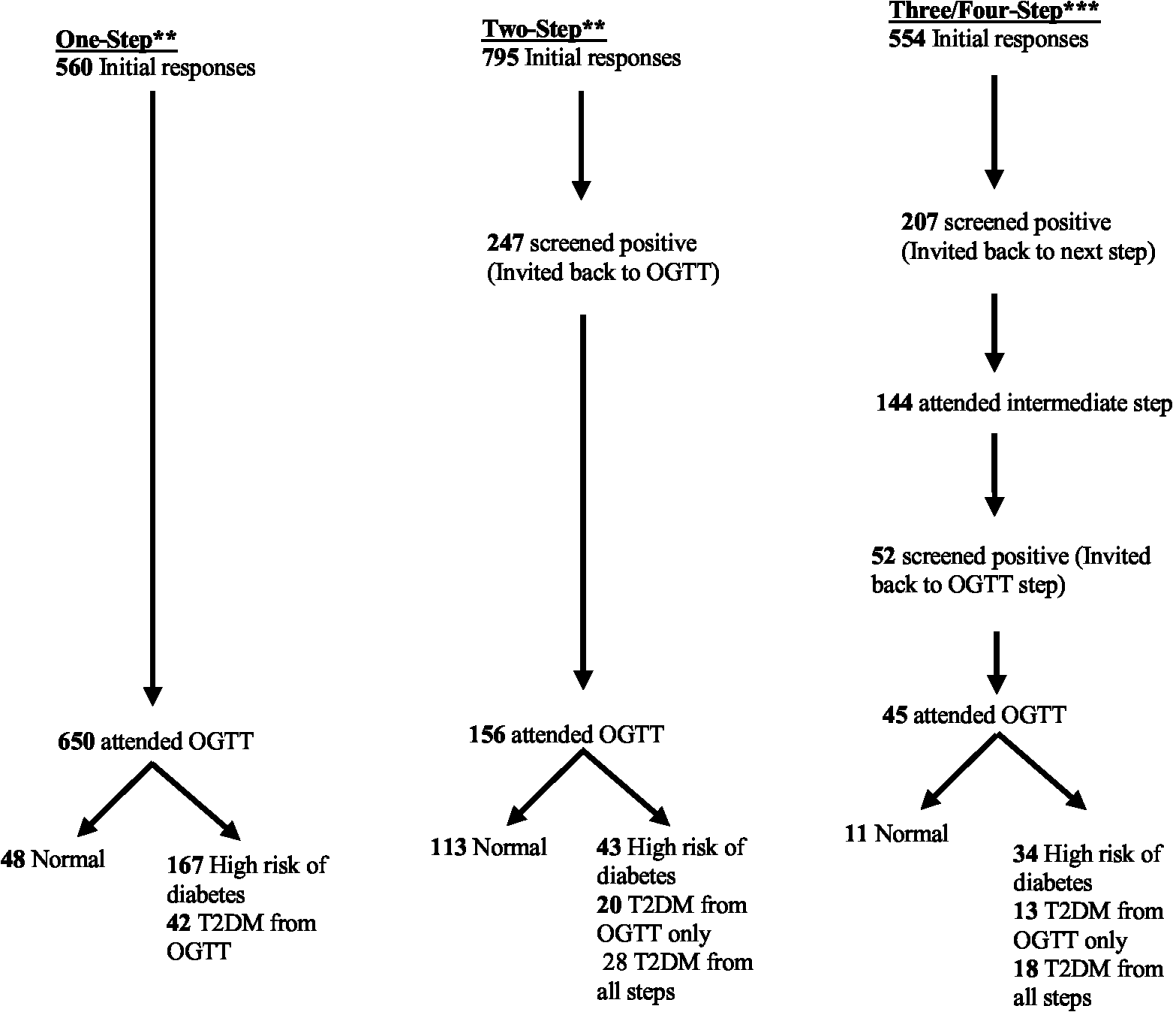
Modelling the screening outcome in a community with 1000 adult population. *One Step strategy = Direct invitation to OGTT (Studies in Table 1). **Two- Step strategy = One step screen before OGTT (Studies in Table 2). ***Three/Four Step Studies = Two or three screening steps before OGTT (Studies in Table 2).

We applied the mean response and yield rates from the meta-analysis to a hypothetical population of 1000 individuals in order to determine the diagnostic yield of the three screening strategies (Fig 5). The number of individuals screened using OGTT decreased from 650 in a one-step strategy to 156 and 45 in two-step and three/four-step screening strategies respectively, and the number of T2DM cases identified decreased from 42 cases in one-step strategy to 28 and 18 in the two-step and three/four-step strategies respectively.

### Invasive versus Non-invasive tests

Seven of the 11 studies with a two-step strategy used an invasive test at the first screening step (Table 2). There was no evidence that the OGTT response rate was higher in studies using an invasive test than those using a non-invasive test [response rate ratio = 1·5, (0·3, 5·7)]. There was a non-significant increase in the yield for T2DM and combined end point of the T2DM and high risk population in studies using invasive test compared to non-invasive methods [yield rate ratio were 6·4 (0·8, 43·9) and 2·3 (0·4, 13·3) respectively]. To compare the initial response rates one study had to be excluded [19] as a computerised risk score was applied instead of sending participants a risk questionnaire. Among the nine studies, there was no evidence to suggest that the initial screening response differed between invasive and non-invasive screening tests [response rate ratio = 4·9 (0·6, 43·7)].

The effect of invasive versus non-invasive tests on the outcomes of the three/four-step strategies was not investigated as 6 of 7 studies employing this strategy used both invasive and non-invasive tests at the intermediate screening steps.

### Other covariate effect and subgroup analyses

Association between screening outcomes and three study-level characteristics: developed versus developing country, invitation method, and urban-rural level are presented in Table 3. There was no evidence that screening and yield rates were associated with a country’s developmental index, the invitation method and the rural or urban location of the community.

**Table 3:** Association between screening outcomes and study level characteristics^†^:- i) developed/developing country^‡^ ii) rural/urban location and iii) invitation method and screening response and yield rates from random effects meta-analysis of T2DM screening studies.

Screening strategy	Study characteristic	Screening response–response rate ratio and 95% CrI		Screening yield–response ratio and 95% CrI	
		OGTT screening	Initial Invitation	OGTT-Diabetes	Diabetes +at risk of diabetes
One step strategy	Developing versus developed country	2.27 (0.80, 6.55)	2.27 (0.80, 6.55)	1.46 (0.93, 2.28)	1.12 (0.74, 1.69)
	Urban versus rural locality	0.94 (0.19, 4.96)	0.85 (0.07, 9.55)	1.81 (0.76, 4.38)	1.53 (0.74, 3.03)
	Letter/Postal invitation versus home visit/face to face invitation	0.80 (0.19, 3.41)	0.52 (0.14, 2.05)	0.90 (0.49, 1.69)	1.03 (0.60, 1.76)
Two step strategy	Developing versus developed country	2.12 (0.55, 8.39)	1.20 (0.14, 9.84)	4.96 (0.69, 32.65)	0.67 (0.08, 5.86)
	Urban versus rural locality	0.17 (0.01, 4.34)	NA	1.81 (0.76, 4.38)	1.21 (0.17, 11.51)
	Letter/Postal invitation versus home visit/face to face invitation	0.44 (0.03, 6.22)	0.09 (0.0, 11.4)	0.046 (0.03, 0.84)	ND

ND = not enough data to carry out analysis. CrI = Credible intervals; Meta-regression analysis was not carried out for three and four step strategy due to lack of data, OGTT = Oral Glucose Tolerance Test

†There was not enough data to perform this analysis for the three/four step studies.

‡Country classes as developed and developing according to United Nations Development Programme (UNDP) Human Development Index (HDI) for 2012. Their classification are shown in Tables 1 and 2.

### Heterogeneity

The estimates of the between-study standard deviation (*τ* on the log-odds scale presented in **S1 Sensitivity Analysis**) indicate a high degree of heterogeneity in rates across studies using a one-step, two-step and three/four-step strategies.

### Sensitivity analysis

Restricting the analysis to studies assessed as good quality (S1 **Quality Assessment**) did not substantially alter pooled OGTT response and yield rates. The effect of study quality was not investigated for the initial and intermediate screening outcomes because of insufficient data at these steps.

In result reported above, we specified the following minimally informative prior distributions: i) Normal (0, 10^3^) for pooled rate on the log-odds scale ii) Uniform (0,100) for the between-study standard deviation. To investigate sensitivity of our results to the choice of prior distributions, we conducted sensitivity analyses for the response and yield outcomes in which we changed the prior distributions to iii) Normal (0, 10^6^) for the pooled rate and ii) Inverse-Gamma (0.001, 0.001) for the between-study variance. The pooled OGTT response rate was 65.2% (53.2% to 75.8%) for one-step strategy, 63.1% (48.2% to 76.9%) for a two-step strategy and 85.1% (78.8% to 91.7) for a three/four-step strategy. These are very similar to main analysis results (Fig 2) suggesting minimal impact of the prior distributions on the pooled response rates. The sensitivity analyses were repeated for the yield outcomes and meta-regression analyses (results not shown) with the same minimal effect on overall pooled rates.

## Discussion

We have summarised and quantified the response and diagnostic yield rates of different screening strategies for T2DM and the high risk population. Our meta-analysis indicates that, for those invited for OGTT screening, the number needed to screen to detect one T2DM case decreases as the number of screening steps increases. We also showed that while the response rates to OGTT invitation is the same for one and two steps screening strategies, the two-step screening study has higher response rate to the initial screening invitation. In the two-step strategies, there was no difference in the response and yield rates whether a blood test was used as the initial screening step or a risk-score. We also showed that the method of invitation did not impact the response rate; whether it was face to face or through post.

The use of risk classification and reclassification is based on the assumption that individuals should be stratified into clinically relevant risk categories and for most risk scores the area under the receiver operating characteristic curve generally range from 0.7 to 0.8, [101] which is seen as an acceptable level of discrimination. Therefore the absolute number of people identified with diabetes or high risk decreases as the number of steps increases (see Fig 3). However, a reduced number of OGTTs are performed to find a higher proportion of people with T2DM or those at high risk. This is both the result of screening out the low risk population and also the number of people who are not responding in each intermediary steps. Ultimately a pragmatic point is that less people need to have OGTT screening when the screening steps increase. This has been suggested to be the most cost effective method [6, 102] and our study confirms the two step methods has higher initial response rate to invitation, similar response rate to OGTT invitation and higher yield rates.

### Comparison with previous studies and recommendations

Screening for a condition is justified only if there is a net benefit in early detection and treatment of the condition as compared to its natural clinical presentation. [103, 104] It has been suggested that T2DM passes this test if a lifestyle intervention is implemented to prevent diabetes in the high risk population. [5, 6] It is argued that universal screening would not be effective or worthwhile, and that populations at risk should be identified and targeted. [105, 106] The impact of screening at population and individual level, costs, type of test to use, and its effectiveness have been highlighted as important factors in decision making [104, 105]. The evidence for management of newly diagnosed diabetes and those at high risk of diabetes for early intervention [107] and prevention [5, 108–111] is compelling. The ADDITION study demonstrated that when compared to routine care, an intervention to promote target-driven, intensive management of patients with type 2 diabetes detected by screening was associated with modest but significant differences in prescribed treatment and levels of cardiovascular risk factors at five years. [112]

The question to answer is the methodology to approach to the screening task. Screening a population using a non-invasive risk stratification tool followed by a definite blood test seems the most cost-effective method of screening for T2DM and those at high risk [6, 102]. Stepwise approach and adding an initial screening test (biochemical or risk score) before the “definitive test” increases the yield from OGTT. Recently National Institute for Clinical Excellence also recommended [6] screening for high risk population and offering blood test for those at high risk of diabetes. Our results confirm this and may have an impact on the cost-effectiveness of this recommendation.

Unfortunately due to lack of qualified studies we were unable to compare screening using different tests such as random or fasting blood glucose, HbA1c, or the risk score as the initial screening step. One study used HbA1c as a screening step [100]. It would also, be ideal if we could report the difference between self-scoring and applied risk score and compare their impact on the response rate to OGTT and overall yield of diabetes.

This is the first meta-analysis to determine response and yield rate when screening for T2DM and people at high risk of diabetes. We used a wide terminology in initial searching for the papers to increase the capture of the publications and reduce missing papers. There was a variety of approaches in invitation and steps prior to the final definitive test (OGTT) and this restricted the meta-analysis and subgroup analysis of the data, however we used robust statistical techniques. We only included the English language papers, but made no limitation on the population size and analysed the data for different regions.

Some studies did not clearly report method of invitation, response rate or exact numbers of participants partaking in certain screening steps. However missing data is always a problem in evidence synthesis and we managed to recover some by contacting authors and or re-calculating the outcomes. Another limitation in any evidence synthesis is the fact that all studies are not really and truly the same and combining their results may not be the real reflection of the pooled outcomes. This is reflected in the high level of heterogeneity seen across all outcomes. In our study; the number and order of the tests used before OGTT, impact of the self-score versus computer generated score, differences between different risk scores, possible impact of population dynamic and the psychosocial factors, wording of the invitation letter and its impact on the response to invitation are examples of some of the possible factors which we could not truly account for their effects on our results.

We used OGTT as the definite test for diagnosis of diabetes. Although, the new guidelines [4, 113] suggest the use of HbA1c for the diagnosis of diabetes, they do not exclude the use of OGTT. Besides, some countries will continue to use OGTT as their screening tool. [12–15] A detailed modelling is necessary to compare the cost-effectiveness of this approach with multi-step screening.

## Conclusion

We have been able to provide evidence for the overall response rate and yield of diabetes screening in the background of a variety of factors such as geographical area, invitation methods and locality of the population (urban vs. rural) which influences decision making when undertaking this task.

We can conclude that performing a multi-step approach in a population screening increases the yield and decreases the number needed to screen by OGTT and in the two- step approach it even increases the initial response rate to the invitation. In terms of absolute numbers, the highest yield of diabetes, however, is obtained in the one step studies where an OGTT is offered as a screening test to the population. The process of screening for T2DM or those at high risk of diabetes needs careful re-evaluation by local policy makers in each country especially in view of our findings.

## Supporting Information

S1 PRISMA Checklist (DOCX)

S1 Quality Assessment (DOCX)

S1 Search Strategies (DOCX)

S1 Sensitivity Analysis (DOCX)

S1 WinBUGS code Random affects meta-analysis. (DOCX)

## References

[1] Whiting DR, Guariguata L, Weil C, Shaw J. IDF diabetes atlas: global estimates of the prevalence of diabetes for 2011 and 2030. Diabetes Res Clin Pract. 2011;94: 311–321. 10.1016/j.diabres.2011.10.029 22079683

[2] DUK. DIABETES IN THE UK 2012 Key statistics on diabetes. April 2012.

[3] Sandbaek A, Griffin SJ, Rutten G, Davies M, Stolk R, Khunti K, et al Stepwise screening for diabetes identifies people with high but modifiable coronary heart disease risk. The ADDITION study. Diabetologia. 2008;51: 1127–1134. 10.1007/s00125-008-1013-0 18443762 PMC2440936

[4] WHO. Use of Glycated Haemoglobin (HbA1c) in the Diagnosis of Diabetes Mellitus available from. 2011;WHO/NMH/CHP/CPM/11.1.

[5] Gillies CL, Lambert PC, Abrams KR, Sutton AJ, Cooper NJ, Hsu RT, et al Different strategies for screening and prevention of type 2 diabetes in adults: cost effectiveness analysis. BMJ. 2008;336: 1180–1185. 10.1136/bmj.39545.585289.25 18426840 PMC2394709

[6] Nice P. Preventing type 2 diabetes: risk identification and interventions for individuals at high risk. 2012.

[7] American Diabetes Association. Diagnosis and classification of diabetes mellitus. Diabetes Care. 2009;32 Suppl 1: S62–7. 10.2337/dc09-S062 19118289 PMC2613584

[8] Wilson JM, Jungner YG. Principles and practice of mass screening for disease. Bol Oficina Sanit Panam. 1968;65: 281–393. 4234760

[9] Skinner TC, Davies MJ, Farooqi AM, Jarvis J, Tringham JR, Khunti K. Diabetes screening anxiety and beliefs. Diabet Med. 2005;22: 1497–1502. 16241913 10.1111/j.1464-5491.2005.01680.x

[10] WHO/IDF. Definition and diagnosis of diabetes mellitus and intermediate hyperglycemia RepoRt of a WHo/IDf ConsultatIon. 2006;(NLM classification: WK 810): ISBN 978 92 4 159493 6.

[11] Lindstrom J, Tuomilehto J. The diabetes risk score: a practical tool to predict type 2 diabetes risk. Diabetes Care. 2003;26: 725–731. 12610029 10.2337/diacare.26.3.725

[12] Herman WH, Cohen RM. Racial and ethnic differences in the relationship between HbA1c and blood glucose: implications for the diagnosis of diabetes. J Clin Endocrinol Metab. 2012;97: 1067–1072. 10.1210/jc.2011-1894 22238408 PMC3319188

[13] John WG, Mosca A, Weykamp C, Goodall I. HbA1c standardisation: history, science and politics. Clin Biochem Rev. 2007;28: 163–168. 18392123 PMC2282401

[14] Gomez-Perez FJ, Aguilar-Salinas CA, Almeda-Valdes P, Cuevas-Ramos D, Lerman Garber I, Rull JA. HbA1c for the diagnosis of diabetes mellitus in a developing country. A position article. Arch Med Res. 2010;41: 302–308. 10.1016/j.arcmed.2010.05.007 20637376

[15] Echouffo-Tcheugui JB, Mayige M, Ogbera AO, Sobngwi E, Kengne AP. Screening for hyperglycemia in the developing world: Rationale, challenges and opportunities. Diabetes Res Clin Pract. 2012;98: 199–208. 10.1016/j.diabres.2012.08.003 22975016

[16] Waugh N, Scotland G, McNamee P, Gillett M, Brennan A, Goyder E, et al Screening for type 2 diabetes: literature review and economic modelling. Health Technol Assess. 2007;11: iii–iv, ix-xi, 1–125. 10.3310/hta11170 17462167

[17] Noble D, Mathur R, Dent T, Meads C, Greenhalgh T. Risk models and scores for type 2 diabetes: systematic review. BMJ. 2011;343: d7163 10.1136/bmj.d7163 22123912 PMC3225074

[18] Rahman M, Simmons RK, Harding AH, Wareham NJ, Griffin SJ. A simple risk score identifies individuals at high risk of developing Type 2 diabetes: a prospective cohort study. Fam Pract. 2008;25: 191–196. 10.1093/fampra/cmn024 18515811

[19] Gray LJ, Khunti K, Edwardson C, Goldby S, Henson J, Morris DH, et al Implementation of the automated Leicester Practice Risk Score in two diabetes prevention trials provides a high yield of people with abnormal glucose tolerance. Diabetologia. 2012;55: 3238–3244. 10.1007/s00125-012-2725-8 23001376

[20] Goyder E, Wild S, Fischbacher C, Carlisle J, Peters J. Evaluating the impact of a national pilot screening programme for type 2 diabetes in deprived areas of England. Fam Pract. 2008;25: 370–375. 10.1093/fampra/cmn054 18765406

[21] [Anonymous]. Randomised controlled trial evaluating cardiovascular screening and intervention in general practice: principal results of British family heart study. Family Heart Study Group. BMJ. 1994;308: 313–320. 8124121 PMC2539278

[22] Jorgensen KJ, Gotzsche PC. Content of invitations for publicly funded screening mammography. BMJ. 2006;332: 538–541. 16513713 10.1136/bmj.332.7540.538 PMC1388137

[23] Marteau TM, Mann E, Prevost AT, Vasconcelos JC, Kellar I, Sanderson S, et al Impact of an informed choice invitation on uptake of screening for diabetes in primary care (DICISION): randomised trial. BMJ. 2010;340: c2138 10.1136/bmj.c2138 20466791 PMC2869404

[24] Eborall HC, Griffin SJ, Prevost AT, Kinmonth AL, French DP, Sutton S. Psychological impact of screening for type 2 diabetes: controlled trial and comparative study embedded in the ADDITION (Cambridge) randomised controlled trial. BMJ. 2007;335: 486 17761995 10.1136/bmj.39303.723449.55 PMC1971192

[25] Eborall H, Stone M, Aujla N, Taub N, Davies M, Khunti K. Influences on the uptake of diabetes screening: a qualitative study in primary care. Br J Gen Pract. 2012;62: e204–11. 10.3399/bjgp12X630106 22429438 PMC3289827

[26] WHO. Definition, Diagnosis and Classification of Diabetes Mellitus and its Complications Report of a WHO Consultation Part 1: Diagnosis and Classification of Diabetes Mellitus. 1999;WHO/NCD/NCS/99.2: Available: https://dl-web.dropbox.com/get/Flash/Avni/WHO%201999.pdf?w=eafbd7d6.

[27] von Elm E, Altman DG, Egger M, Pocock SJ, Gotzsche PC, Vandenbroucke JP, et al The Strengthening the Reporting of Observational Studies in Epidemiology (STROBE) statement: guidelines for reporting observational studies. J Clin Epidemiol. 2008;61: 344–349. 10.1016/j.jclinepi.2007.11.008 18313558

[28] Wong WC, Cheung CS, Hart GJ. Development of a quality assessment tool for systematic reviews of observational studies (QATSO) of HIV prevalence in men having sex with men and associated risk behaviours. Emerg Themes Epidemiol. 2008;5: 23 10.1186/1742-7622-5-23 19014686 PMC2603000

[29] Spiegelhalter D, Thomas A, Best N, Lunn D. WinBUGS user manual version 1.4 January 2003. Upgraded to Version 1.4.3. Available from.

[30] Al-Lawati JA, Tuomilehto J. Diabetes risk score in Oman: a tool to identify prevalent type 2 diabetes among Arabs of the Middle East. Diabetes Res Clin Pract 2007 9;77(3):438–444. 17306410 10.1016/j.diabres.2007.01.013

[31] Egeland GM, Cao Z, Young TK. Hypertriglyceridemic-waist phenotype and glucose intolerance among Canadian Inuit: the International Polar Year Inuit Health Survey for Adults 2007–2008. CMAJ 2011 6 14;183(9):E553–8. 10.1503/cmaj.101801 21555386 PMC3114931

[32] Goyder E, Wild S, Fischbacher C, Carlisle J, Peters J. Evaluating the impact of a national pilot screening programme for type 2 diabetes in deprived areas of England. Fam Pract 2008 10;25(5):370–375. 10.1093/fampra/cmn054 18765406

[33] Mensink M, Corpeleijn E, Feskens EJ, Kruijshoop M, Saris WH, de Bruin TW, et al Study on lifestyle-intervention and impaired glucose tolerance Maastricht (SLIM): design and screening results. Diabetes Res Clin Pract 2003 7;61(1):49–58. 12849923 10.1016/s0168-8227(03)00067-6

[34] Oberlinner C, Neumann SM, Ott MG, Zober A. Screening for pre-diabetes and diabetes in the workplace. Occup Med (Lond) 2008 1;58(1):41–45. 18025060 10.1093/occmed/kqm129

[35] Saaristo T, Peltonen M, Lindstrom J, Saarikoski L, Sundvall J, Eriksson JG, et al Cross-sectional evaluation of the Finnish Diabetes Risk Score: a tool to identify undetected type 2 diabetes, abnormal glucose tolerance and metabolic syndrome. Diab Vasc Dis Res 2005 5;2(2):67–72. 16305061 10.3132/dvdr.2005.011

[36] Sadeghi M, Roohafza H, Shirani S, Poormoghadas M, Kelishadi R, Baghaii A, et al Diabetes and associated cardiovascular risk factors in Iran: the Isfahan Healthy Heart Programme. Ann Acad Med Singapore 2007 3;36(3):175–180. 17450262

[37] Sundborn G, Metcalf P, Scragg R, Schaaf D, Dyall L, Gentles D, et al Ethnic differences in the prevalence of new and known diabetes mellitus, impaired glucose tolerance, and impaired fasting glucose. Diabetes Heart and Health Survey (DHAH) 2002–2003, Auckland New Zealand. N Z Med J 2007 6 29;120(1257):U2607 17632597

[38] Ziemer DC, Kolm P, Foster JK, Weintraub WS, Vaccarino V, Rhee MK, et al Random plasma glucose in serendipitous screening for glucose intolerance: screening for impaired glucose tolerance study 2. J Gen Intern Med 2008 5;23(5):528–535. 10.1007/s11606-008-0524-1 18335280 PMC2324161

[39] Ziemer DC, Kolm P, Weintraub WS, Vaccarino V, Rhee MK, Twombly JG, et al Glucose-independent, black-white differences in hemoglobin A1c levels: a cross-sectional analysis of 2 studies. Ann Intern Med 2010 6 15;152(12):770–777. 10.7326/0003-4819-152-12-201006150-00004 20547905

[40] Azizi F, Gouya MM, Vazirian P, Dolatshahi P, Habibian S. The diabetes prevention and control programme of the Islamic Republic of Iran. East Mediterr Health J 2003 Sep-Nov;9(5–6):1122–1127. 16450545

[41] Mannucci E, Ognibene A, Sposato I, Brogi M, Gallori G, Bardini G, et al Fasting plasma glucose and glycated haemoglobin in the screening of diabetes and impaired glucose tolerance. Acta Diabetol. 2003;40: 181–186. 14740278 10.1007/s00592-003-0109-8

[42] Jia WP, Pang C, Chen L, Bao YQ, Lu JX, Lu HJ, et al Epidemiological characteristics of diabetes mellitus and impaired glucose regulation in a Chinese adult population: the Shanghai Diabetes Studies, a cross-sectional 3-year follow-up study in Shanghai urban communities. Diabetologia. 2007;50: 286–292. 17180353 10.1007/s00125-006-0503-1

[43] Das M, Hassan Z, Faruque O, Parial R, Khalequzzaman M, Ali L. Prevalence of abnormal glycemic and lipidemic status in an urban population of Bangladesh. J bio-sci. 2011;19: 1–6.

[44] Habibi Moeine S, Mirmiran P, Mehrabi Y, Azizi F. Evaluation of Different Risk Factors for Early Diagnosis of Diabetes Mellitus. IJMS. 2004;29: 21–25.

[45] Franciosi M, De Berardis G, Rossi MC, Sacco M, Belfiglio M, Pellegrini F, et al Use of the diabetes risk score for opportunistic screening of undiagnosed diabetes and impaired glucose tolerance: the IGLOO (Impaired Glucose Tolerance and Long-Term Outcomes Observational) study. Diabetes Care. 2005;28: 1187–1194. 15855587 10.2337/diacare.28.5.1187

[46] Zhou X, Ji L, Luo Y, Han X, Zhang X, Sun X, et al Risk factors associated with the presence of diabetes in Chinese communities in Beijing. Diabetes Res Clin Pract. 2009;86: 233–238. 10.1016/j.diabres.2009.09.014 19836096

[47] Zhou XH, Ji LN, Luo YY, Zhang XY, Han XY, Qiao Q. Performance of HbA(1c) for detecting newly diagnosed diabetes and pre-diabetes in Chinese communities living in Beijing. Diabet Med. 2009;26: 1262–1268. 10.1111/j.1464-5491.2009.02831.x 20002479

[48] Rush E, Crook N, Simmons D. Point-of-care testing as a tool for screening for diabetes and pre-diabetes. Diabet Med. 2008;25: 1070–1075. 10.1111/j.1464-5491.2008.02526.x 19183312

[49] El Bassuoni EA, Ziemer DC, Kolm P, Rhee MK, Vaccarino V, Tsui CW, et al The "metabolic syndrome" is less useful than random plasma glucose to screen for glucose intolerance. Prim Care Diabetes. 2008;2: 147–153. 10.1016/j.pcd.2008.04.005 18779039 PMC2638987

[50] Riste L, Khan F, Cruickshank K. High prevalence of type 2 diabetes in all ethnic groups, including Europeans, in a British inner city: relative poverty, history, inactivity, or 21st century Europe? Diabetes Care. 2001;24: 1377–1383. 11473073 10.2337/diacare.24.8.1377

[51] Amoah AG, Owusu SK, Adjei S. Diabetes in Ghana: a community based prevalence study in Greater Accra. Diabetes Res Clin Pract. 2002;56: 197–205. 11947967 10.1016/s0168-8227(01)00374-6

[52] Botas P, Delgado E, Castano G, Diaz de Grenu C, Prieto J, Diaz-Cadorniga FJ. Comparison of the diagnostic criteria for diabetes mellitus, WHO-1985, ADA-1997 and WHO-1999 in the adult population of Asturias (Spain). Diabet Med. 2003;20: 904–908. 14632715 10.1046/j.1464-5491.2003.01006.x

[53] Rathmann W, Haastert B, Icks A, Lowel H, Meisinger C, Holle R, et al High prevalence of undiagnosed diabetes mellitus in Southern Germany: target populations for efficient screening. The KORA survey 2000. Diabetologia. 2003;46: 182–189. 12627316 10.1007/s00125-002-1025-0

[54] Colagiuri S, Hussain Z, Zimmet P, Cameron A, Shaw J, AusDiab. Screening for type 2 diabetes and impaired glucose metabolism: the Australian experience. Diabetes Care. 2004;27: 367–371. 14747215 10.2337/diacare.27.2.367

[55] Glumer C, Jorgensen T, Borch-Johnsen K. Targeted screening for undiagnosed diabetes reduces the number of diagnostic tests. Inter99(8). Diabet Med. 2004;21: 874–880. 15270791 10.1111/j.1464-5491.2004.01260.x

[56] Glumer C, Jorgensen T, Borch-Johnsen K, Inter99 study. Prevalences of diabetes and impaired glucose regulation in a Danish population: the Inter99 study. Diabetes Care. 2003;26: 2335–2340. 12882858 10.2337/diacare.26.8.2335

[57] Simmons D, McKenzie A, Eaton S, Shaw J, Zimmet P. Prevalence of diabetes in rural Victoria. Diabetes Res Clin Pract. 2005;70: 287–290. 15946759 10.1016/j.diabres.2005.04.004

[58] Brohall G, Behre CJ, Hulthe J, Wikstrand J, Fagerberg B. Prevalence of diabetes and impaired glucose tolerance in 64-year-old Swedish women: experiences of using repeated oral glucose tolerance tests. Diabetes Care. 2006;29: 363–367. 16443888 10.2337/diacare.29.02.06.dc05-1229

[59] Heldgaard PE, Griffin SJ. Routinely collected general practice data aids identification of people with hyperglycaemia and metabolic syndrome. Diabet Med. 2006;23: 996–1002. 16922706 10.1111/j.1464-5491.2006.01929.x

[60] Heldgaard PE, Henriksen JE, Sidelmann JJ, Olivarius Nde F, Siersma VD, Gram JB. Similar cardiovascular risk factor profile in screen-detected and known type 2 diabetic subjects. Scand J Prim Health Care. 2011;29: 85–91. 10.3109/02813432.2011.565164 21438763 PMC3347946

[61] Menon VU, Kumar KV, Gilchrist A, Sugathan TN, Sundaram KR, Nair V, et al Prevalence of known and undetected diabetes and associated risk factors in central Kerala—ADEPS. Diabetes Res Clin Pract. 2006;74: 289–294. 16730847 10.1016/j.diabres.2006.03.025

[62] Mohan V, Deepa M, Deepa R, Shanthirani CS, Farooq S, Ganesan A, et al Secular trends in the prevalence of diabetes and impaired glucose tolerance in urban South India—the Chennai Urban Rural Epidemiology Study (CURES-17). Diabetologia. 2006;49: 1175–1178. 16570158 10.1007/s00125-006-0219-2

[63] Mohan V, Sandeep S, Deepa M, Gokulakrishnan K, Datta M, Deepa R. A diabetes risk score helps identify metabolic syndrome and cardiovascular risk in Indians—the Chennai Urban Rural Epidemiology Study (CURES-38). Diabetes Obes Metab. 2007;9: 337–343. 17391160 10.1111/j.1463-1326.2006.00612.x

[64] Deepa M, Farooq S, Datta M, Deepa R, Mohan V. Prevalence of metabolic syndrome using WHO, ATPIII and IDF definitions in Asian Indians: the Chennai Urban Rural Epidemiology Study (CURES-34). Diabetes Metab Res Rev. 2007;23: 127–134. 16752431 10.1002/dmrr.658

[65] Barakat MN, Youssef RM. Prevalence of dysglycemia and other cardiovascular risk factors among the rural population of Oman. Saudi Med J. 2008;29: 1824–1826. 19082242

[66] Karjalainen J, Peltonen M, Vanhala M, Korpi-Hyovalti E, Puolijoki H, Saltevo J, et al Leisure time physical activity in individuals with screen-detected type 2 diabetes compared to those with known type 2 diabetes. Diabetes Res Clin Pract. 2008;81: 110–1166. 10.1016/j.diabres.2008.03.006 18433914

[67] Bener A, Zirie M, Janahi IM, Al-Hamaq AO, Musallam M, Wareham NJ. Prevalence of diagnosed and undiagnosed diabetes mellitus and its risk factors in a population-based study of Qatar. Diabetes Res Clin Pract. 2009;84: 99–106 10.1016/j.diabres.2009.02.003 19261345

[68] Dunkley AJ, Taub NA, Davies MJ, Stone MA, Khunti K. Is having a family history of type 2 diabetes or cardiovascular disease a predictive factor for metabolic syndrome? Prim Care Diabetes. 2009;3: 49–56. 10.1016/j.pcd.2009.02.002 19268647

[69] Gardete-Correia L, Boavida JM, Raposo JF, Mesquita AC, Fona C, Carvalho R, et al First diabetes prevalence study in Portugal: PREVADIAB study. Diabet Med. 2010;27: 879–881. 10.1111/j.1464-5491.2010.03017.x 20653744

[70] Cho NH, Jang HC, Choi SH, Kim HR, Lee HK, Chan JC, et al Abnormal liver function test predicts type 2 diabetes: a community-based prospective study. Diabetes Care. 2007;30: 2566–2568. 17626893 10.2337/dc07-0106

[71] Choi SH, Kim TH, Lim S, Park KS, Jang HC, Cho NH. Hemoglobin A1c as a Diagnostic Tool for Diabetes Screening and New-Onset Diabetes Prediction. Diabetes care. 2011;34: 944–949. 10.2337/dc10-0644 21335372 PMC3064055

[72] Choi SJ, Keam B, Park SH, Park HY. Appropriate waist circumference cut-offs to predict diabetes in the Korean population—the Korean Genome and Epidemiology Study-. Circ J. 2010;74: 1357–1363. 20519877 10.1253/circj.cj-09-0739

[73] Lim S, Jang HC, Lee HK, Kimm KC, Park C, Cho NH. A rural-urban comparison of the characteristcs of the metabolic syndrome by gender in korea: The Korean Health and Genome Study (KHGS). J Endocrinol Invest. 2006;29: 313–319. 16699297 10.1007/BF03344102

[74] Webb DR, Gray LJ, Khunti K, Srinivasan B, Taub N, Campbell S, et al Screening for diabetes using an oral glucose tolerance test within a western multi-ethnic population identifies modifiable cardiovascular risk: the ADDITION-Leicester study. Diabetologia. 2011;54: 2237–2246. 10.1007/s00125-011-2189-2 21638133

[75] Bhansali A, Walia R, Ravi Kumar P, Ravi Kiran M, Shanmugasundar G. Accuracy of glycated haemoglobin in screening for pre-diabetes in Asian Indians—a community survey: the Chandigarh Urban Diabetes Study (CUDS). Diabet Med. 2012;29: 1385–1389. 10.1111/j.1464-5491.2012.03634.x 22414322

[76] Hayes L, Hawthorne G, Unwin N. Undiagnosed diabetes in the over-60s: performance of the Association of Public Health Observatories (APHO) Diabetes Prevalence Model in a general practice. Diabet Med. 2012;29: 115–120. 10.1111/j.1464-5491.2011.03389.x 21781154

[77] Ye Z, Cong L, Ding G, Yu M, Zhang X, Hu R, et al (2014) Optimal Cut-Off Points for Two-Step Strategy in Screening of Undiagnosed Diabetes: A Population- Based Study in China. PLoS ONE 9(3): e87690 10.1371/journal.pone.0087690 24609110 PMC3946449

[78] Dong Y, Gao W, Nan H, Yu H, Li F, Duan W, et al Prevalence of Type 2 diabetes in urban and rural Chinese populations in Qingdao, China. Diabet Med. 2005;22: 1427–1433. 16176207 10.1111/j.1464-5491.2005.01658.x

[79] Eriksson AK, Ekbom A, Hilding A, Ostenson CG. The influence of non-response in a population-based cohort study on type 2 diabetes evaluated by the Swedish Prescribed Drug Register. Eur J Epidemiol. 2012;27: 153–162. 10.1007/s10654-011-9630-1 22089424

[80] Hilding A, Eriksson AK, Agardh EE, Grill V, Ahlbom A, Efendic S, et al The impact of family history of diabetes and lifestyle factors on abnormal glucose regulation in middle-aged Swedish men and women. Diabetologia. 2006;49: 2589–2598. 16969647 10.1007/s00125-006-0402-5

[81] Korhonen PE, Jaatinen PT, Aarnio PT, Kantola IM, Saaresranta T. Waist circumference home measurement—a device to find out patients in cardiovascular risk. Eur J Public Health. 2009;19: 95–99. 10.1093/eurpub/ckn090 18927187

[82] Ohkura T, Taniguchi S, Inoue K, Yamamoto N, Matsuzawa K, Fujioka Y, et al Screening Criteria of Diabetes Mellitus and Impaired Glucose Tolerance of the Japanese Population in a Rural Area of Japan: The Tottori-Kofu Study. Yonago Acta medica. 2009;52: 105–114.

[83] Rahim MA, Vaaler S, Keramat Ali SM, Khan AK, Hussain A, Nahar Q. Prevalence of type 2 diabetes in urban slums of Dhaka, Bangladesh. Bangladesh Med Res Counc Bull. 2004;30: 60–70. 15813484

[84] Rahim MA, Hussain A, Azad Khan AK, Sayeed MA, Keramat Ali SM, Vaaler S. Rising prevalence of type 2 diabetes in rural Bangladesh: a population based study. Diabetes Res Clin Pract. 2007;77: 300–305. 17187890 10.1016/j.diabres.2006.11.010

[85] Rahim MA, Azad Khan AK, Nahar Q, Ali SM, Hussain A. Impaired fasting glucose and impaired glucose tolerance in rural population of Bangladesh. Bangladesh Med Res Counc Bull. 2010;36: 47–51. 21473200 10.3329/bmrcb.v36i2.6986

[86] Hussain A, Rahim MA, Azad Khan AK, Ali SM, Vaaler S. Type 2 diabetes in rural and urban population: diverse prevalence and associated risk factors in Bangladesh. Diabet Med. 2005;22: 931–936. 15975110 10.1111/j.1464-5491.2005.01558.x

[87] Makrilakis K, Liatis S, Grammatikou S, Perrea D, Stathi C, Tsiligros P, et al Validation of the Finnish diabetes risk score (FINDRISC) questionnaire for screening for undiagnosed type 2 diabetes, dysglycaemia and the metabolic syndrome in Greece. Diabetes Metab. 2011;37: 144–151. 10.1016/j.diabet.2010.09.006 21144787

[88] Lin S, Hu L, Li X, Chen Y, Xu H, He S, et al Glycated haemoglobin A c for diagnosing diabetes in Chinese subjects over 50 years old: a community-based cross-sectional study. Clin Endocrinol (Oxf). 2013. 10.1111/cen.12202 23488681

[89] Phillips LS, Ziemer DC, Kolm P, Weintraub WS, Vaccarino V, Rhee MK. et al Glucose challenge test screening for prediabetes and undiagnosed diabetes Diabetologia (2009) 52:1798–1807 10.1007/s00125-009-1407-7 19557386

[90] Spijkerman AM, Adriaanse MC, Dekker JM, Nijpels G, Stehouwer CD, Bouter LM, et al Diabetic patients detected by population-based stepwise screening already have a diabetic cardiovascular risk profile. Diabetes Care. 2002;25: 1784–1789. 12351478 10.2337/diacare.25.10.1784

[91] Sandbaek A, Lauritzen T, Borch-Johnsen K, Mai K, Christiansen JS. The comparison of venous plasma glucose and whole blood capillary glucose in diagnoses of Type 2 diabetes: a population-based screening study. Diabet Med. 2005;22: 1173–1177. 16108845 10.1111/j.1464-5491.2005.01491.x

[92] Dalsgaard EM, Christensen JO, Skriver MV, Borch-Johnsen K, Lauritzen T, Sandbaek A. Comparison of different stepwise screening strategies for type 2 diabetes: Finding from Danish general practice, Addition-DK. Prim Care Diabetes. 2010;4: 223–229. 10.1016/j.pcd.2010.06.003 20675208

[93] Janssen PG, Gorter KJ, Stolk RP, Akarsubasi M, Rutten GE. Three years follow-up of screen-detected diabetic and non-diabetic subjects: who is better off? The ADDITION Netherlands study. BMC Fam Pract. 2008;9: 67 10.1186/1471-2296-9-67 19087327 PMC2626593

[94] Janssen PG, Gorter KJ, Stolk RP, Rutten GE. Screen detected subjects with type 2 diabetes and impaired glucose tolerance have more adverse cardiovascular risk than subjects with impaired fasting glucose especially when they are obese: the ADDITION Netherlands study. Prim Care Diabetes. 2007;1: 69–74. 10.1016/j.pcd.2007.02.001 18632022

[95] Janssen PG, Gorter KJ, Stolk RP, Rutten GE. Low yield of population-based screening for Type 2 diabetes in the Netherlands: the ADDITION Netherlands study. Fam Pract. 2007;24: 555–561. 17962235 10.1093/fampra/cmm052

[96] Christensen JO, Sandbaek A, Lauritzen T, Borch-Johnsen K. Population-based stepwise screening for unrecognised Type 2 diabetes is ineffective in general practice despite reliable algorithms. Diabetologia. 2004;47: 1566–1573. 15365615 10.1007/s00125-004-1496-2

[97] Sargeant LA, Simmons RK, Barling RS, Butler R, Williams KM, Prevost AT, et al Who attends a UK diabetes screening programme? Findings from the ADDITION-Cambridge study. Diabet Med. 2010;27: 995–1003. 10.1111/j.1464-5491.2010.03056.x 20722672 PMC3428846

[98] Park P, Simmons RK, Prevost AT, Griffin SJ. Screening for type 2 diabetes is feasible, acceptable, but associated with increased short-term anxiety: a randomised controlled trial in British general practice. BMC Public Health. 2008;8: 350 10.1186/1471-2458-8-350 18840266 PMC2567326

[99] Park P, Simmons RK, Prevost AT, Griffin SJ, ADDITION Cambridge study group. A randomized evaluation of loss and gain frames in an invitation to screening for type 2 diabetes: effects on attendance, anxiety and self-rated health. J Health Psychol. 2010;15: 196–204. 10.1177/1359105309344896 20207663

[100] McAullay D, Sibthorpe B, Knuiman M. Evaluation of a new diabetes screening method at the Derbarl Yerrigan Health Service. Aust N Z J Public Health. 2004;28: 43–46. 15108746 10.1111/j.1467-842x.2004.tb00631.x

[101] Buijsse B, Simmons RK, Griffin SJ, Schulze MB. Risk assessment tools for identifying individuals at risk of developing type 2 diabetes. Epidemiol Rev. 2011;33: 46–62. 10.1093/epirev/mxq019 21622851 PMC3132807

[102] Khunti K, Gillies CL, Taub NA, Mostafa SA, Hiles SL, Abrams KR, et al A comparison of cost per case detected of screening strategies for Type 2 diabetes and impaired glucose regulation: Modelling study. Diabetes Res Clin Pract. 2012;97: 505–513. 10.1016/j.diabres.2012.03.009 22554999

[103] Harris R, Donahue K, Rathore SS, Frame P, Woolf SH, Lohr KN. Screening adults for type 2 diabetes: a review of the evidence for the U.S. Preventive Services Task Force. Ann Intern Med. 2003;138: 215–229. 12558362 10.7326/0003-4819-138-3-200302040-00015

[104] Norris SL, Kansagara D, Bougatsos C, Fu R, U.S. Preventive Services Task Force. Screening adults for type 2 diabetes: a review of the evidence for the U.S. Preventive Services Task Force. Ann Intern Med. 2008;148: 855–868. 18519931 10.7326/0003-4819-148-11-200806030-00008

[105] WHO I. Screening for Type 2 Diabetes Report of a World Health Organization and International Diabetes Federation meeting. 2003;WHO/NMH/MNC/03.1 Available: http://www.who.int/diabetes/publications/en/screening_mnc03.pdf.

[106] Wareham NJ, Griffin SJ. Should we screen for type 2 diabetes? Evaluation against National Screening Committee criteria. BMJ. 2001;322: 986–988. 11312236 10.1136/bmj.322.7292.986 PMC1120142

[107] Holman RR, Paul SK, Bethel MA, Matthews DR, Neil HA. 10-Year Follow-Up of Intensive Glucose Control in Type 2 Diabetes. N Engl J Med. 2008;359: 1577–1589. 10.1056/NEJMoa0806470 18784090

[108] Diabetes Prevention Program Research Group, Knowler WC, Fowler SE, Hamman RF, Christophi CA, Hoffman HJ, et al 10-year follow-up of diabetes incidence and weight loss in the Diabetes Prevention Program Outcomes Study. Lancet. 2009;374: 1677–1686. 10.1016/S0140-6736(09)61457-4 19878986 PMC3135022

[109] Lindstrom J, Ilanne-Parikka P, Peltonen M, Aunola S, Eriksson JG, Hemio K, et al Sustained reduction in the incidence of type 2 diabetes by lifestyle intervention: follow-up of the Finnish Diabetes Prevention Study. Lancet. 2006;368: 1673–1679. 17098085 10.1016/S0140-6736(06)69701-8

[110] Khunti K, Davies MJ. Diabetes prevention: NICE opportunity for implementing programmes in the real-world setting. Diabet Med. 2012. 10.1111/dme.12042 23072427

[111] Khunti K, Davies M. Should we screen for type 2 diabetes: Yes. BMJ. 2012;345: e4514 10.1136/bmj.e4514 22777029

[112] Simmons RK, Echouffo-Tcheugui JB, Sharp SJ, Sargeant LA, Williams KM, Prevost AT, et al Screening for type 2 diabetes and population mortality over 10 years (ADDITION-Cambridge): a cluster-randomised controlled trial. Lancet. 2012. 10.1016/S0140-6736(12)61422-6 PMC3607818 23040422

[113] John WG, on behalf of the UK Department of Health Advisory Committee on Diabetes. Use of HbA(1c) in the diagnosis of diabetes mellitus in the UK. The implementation of World Health Organization guidance 2011. Diabet Med. 2012;29: 1350–1357. 10.1111/j.1464-5491.2012.03762.x 22957983

