# Supplementary material for: Systematic Review and Meta-Analysis of Response Rates and Diagnostic Yield of Screening for Type 2 Diabetes and Those at High Risk of Diabetes
Source: PLoS One. 2015 Sep 1;10(9):e0135702. doi: 10.1371/journal.pone.0135702 (PMC4556656; doi:10.1371/journal.pone.0135702)
Supplement: S1 Quality Assessment — (DOCX) [file pone.0135702.s002.docx]

**S1 Quality Assessment**

1. Is the study aim clear?
2. Is the sampling method representative of the whole population?
3. Was it a random selection of the participants?
4. Participant recruitment: Was inclusion/exclusion criteria described?
5. Was it community setting?
6. Did the study report response rate?
7. Results are clear of to how many people were diagnosed with IGT IFG T2D (e.g prevalence, percentages or yield)
8. Were withdrawals/missing data explained?
9. Have they reported Confidence Interval/P value?

| **0% -33·9%** | **34%- 66·9%** | **67% -100%** |
| --- | --- | --- |
| **Poor** | **Satisfactory** | **Good** |

Table A: Quality of studies assessment

|  | **Score** | **Q1** | **Q2** | **Q3** | **Q4** | **Q5** | **Q6** | **Q7** | **Q8** | **Q9** |
| --- | --- | --- | --- | --- | --- | --- | --- | --- | --- | --- |
| Amoah (2002) | **8** | 1 | 1 | 1 | 1 | 1 | 0 | 1 | 1 | 1 |
| Barakat (2008) | **8** | 1 | 1 | 1 | 1 | 1 | 1 | 1 | 1 | 0 |
| Bener (2009) | **8** | 1 | 1 | 1 | 1 | 1 | 1 | 1 | 1 | 0 |
| Botas (2003) | **7** | 1 | 0 | 1 | 1 | 1 | 1 | 1 | 1 | 0 |
| Brohall (2006) | **7** | 1 | 1 | 1 | 1 | 1 | 1 | 1 | 0 | 0 |
| Choi (2011) | **8** | 1 | 1 | 1 | 1 | 1 | 1 | 1 | 0 | 1 |
| Christensen (2004) | **8** | 1 | 1 | 1 | 1 | 1 | 1 | 1 | 1 | 0 |
| Colagirui (2004) | **8** | 1 | 1 | 1 | 1 | 1 | 1 | 1 | 1 | 0 |
| Correia (2010) | **7** | 1 | 1 | 1 | 1 | 1 | 1 | 0 | 0 | 1 |
| Dong (2005) | **7** | 1 | 1 | 1 | 1 | 1 | 1 | 1 | 0 | 0 |
| Dunkley (2009) | **6** | 1 | 1 | 1 | 1 | 1 | 0 | 1 | 0 | 0 |
| El Bassuoni (2008) | **4** | 1 | 0 | 0 | 1 | 0 | 0 | 1 | 1 | 0 |
| Franciosi (2005) | **5** | 1 | 0 | 0 | 1 | 1 | 0 | 1 | 1 | 0 |
| Glumer (2004) | **8** | 1 | 1 | 1 | 1 | 1 | 1 | 1 | 1 | 0 |
| Habibi M (2004) | **5** | 1 | 1 | 1 | 1 | 0 | 0 | 1 | 0 | 0 |
| Heldgaard (2006) | **9** | 1 | 1 | 1 | 1 | 1 | 1 | 1 | 1 | 1 |
| Janssen I (2007) | **6** | 1 | 1 | 1 | 0 | 1 | 0 | 1 | 1 | 0 |
| Janssen II (2007) | **7** | 1 | 1 | 1 | 1 | 1 | 0 | 1 | 0 | 1 |
| Jia (2007) | **8** | 1 | 1 | 1 | 1 | 1 | 0 | 1 | 1 | 1 |
| Karjalainen (2008) | **8** | 1 | 1 | 1 | 1 | 1 | 1 | 1 | 1 | 0 |
| Korhonen (2008) | **7** | 0 | 1 | 1 | 1 | 1 | 1 | 1 | 1 | 0 |
| Makrilakis (2011) | **7** | 1 | 1 | 1 | 1 | 1 | 1 | 0 | 0 | 1 |
| Mannucci (2003) | **3** | 1 | 0 | 0 | 1 | 0 | 0 | 1 | 0 | 0 |
| Mcaullay (2004) | **5** | 1 | 0 | 0 | 1 | 1 | 0 | 1 | 1 | 0 |
| Menon (2006) | **8** | 1 | 1 | 1 | 1 | 1 | 1 | 1 | 0 | 1 |
| Mohan (2006) | **8** | 1 | 1 | 1 | 1 | 1 | 1 | 1 | 1 | 0 |
| Ohkura (2009) | **5** | 1 | 0 | 0 | 0 | 1 | 1 | 1 | 1 | 0 |
| Park (2010) | **7** | 1 | 1 | 1 | 1 | 1 | 1 | 0 | 0 | 1 |
| Rahim (2010) | **8** | 1 | 1 | 1 | 1 | 1 | 1 | 1 | 0 | 1 |
| Rathman (2003) | **9** | 1 | 1 | 1 | 1 | 1 | 1 | 1 | 1 | 1 |
| Riste (2001) | **9** | 1 | 1 | 1 | 1 | 1 | 1 | 1 | 1 | 1 |
| Rush (2008) | **6** | 1 | 1 | 0 | 1 | 1 | 0 | 1 | 1 | 0 |
| Sandbaek (2005) | **8** | 1 | 1 | 1 | 1 | 1 | 1 | 1 | 1 | 0 |
| Sargeant (2010) | **9** | 1 | 1 | 1 | 1 | 1 | 1 | 1 | 1 | 1 |
| Simmons (2005) | **9** | 1 | 1 | 1 | 1 | 1 | 1 | 1 | 1 | 1 |
| Spikjerman (2002) | **8** | 1 | 1 | 1 | 1 | 1 | 1 | 1 | 1 | 0 |
| Webb (2011) | **8** | 1 | 1 | 1 | 1 | 1 | 0 | 1 | 1 | 1 |
| Zhou (2009) | **6** | 1 | 0 | 0 | 1 | 1 | 0 | 1 | 1 | 1 |
| Bhansali (2012) | **7** | 1 | 1 | 1 | 0 | 1 | 1 | 1 | 0 | 1 |
| Hilding (2012) | **8** | 1 | 1 | 1 | 1 | 1 | 1 | 1 | 1 | 0 |
| Gray (2012) | **8** | 1 | 1 | 0 | 1 | 1 | 1 | 1 | 1 | 1 |
| Hayes (2012) | **8** | 1 | 1 | 1 | 1 | 1 | 1 | 1 | 0 | 1 |
| Yu (2012) | **6** | 1 | 0 | 0 | 1 | 1 | 0 | 1 | 1 | 1 |
| Lin (2013) | **8** | 1 | 1 | 1 | 1 | 1 | 1 | 1 | 1 | 0 |
| Das (2011) | **3** | 1 | 0 | 0 | 0 | 1 | 0 | 1 | 0 | 0 |
| Phillips (2009) | **3** | 1 | 0 | 0 | 1 | 1 | 0 | 0 | 0 | 0 |
| Ye (2014) | **5** | 1 | 1 | 1 | 0 | 1 | 1 | 0 | 0 | 0 |
