# Supplementary material for: Systematic Review and Meta-Analysis of Response Rates and Diagnostic Yield of Screening for Type 2 Diabetes and Those at High Risk of Diabetes
Source: PLoS One. 2015 Sep 1;10(9):e0135702. doi: 10.1371/journal.pone.0135702 (PMC4556656; doi:10.1371/journal.pone.0135702)
Supplement: S1 Search Strategies — (DOCX) [file pone.0135702.s003.docx]

**Supporting Information**

**S1 Search Strategies**

Medline and Embase:

1. Exp Diabetes Mellitus, Type 2/
2. Diabetes.ti,ab
3. Prediabetes.ti,ab
4. Pre-diabetes.ti,ab
5. Exp prediabetic state/
6. Exp Glucose Intolerance/
7. Exp Hyperglycemia/
8. (impaired adj2 glucose adj2 regulation) ti,ab
9. Impaired glucose tolerance.ti,ab
10. Impaired fasting glyc?emia.ti,ab
11. Dysglyc?emia.ti,ab
12. 6 or 11 or 3 or 7 or 9 or 2 or 8 or 1 or 4 or 10 or 5
13. Screening.ti,ab
14. Exp Mass screening/
15. Exp Early Diagnosis/
16. (Early adj2 detect$).ti,ab
17. 16 or 13 or 14 or 15
18. Letter.pt.
19. Case report/
20. Editorial.pt
21. 18 or 19 or 20
22. 17 AND 12
23. 22 not 21
24. Limit 23 to humans
25. Limit 24 to yr=”1998-current”

Cochrane:

1. MeSH descriptor Diabetes Mellitus explode all trees
2. (diabetes):ti,ab,kw
3. (prediabetes):ti,ab,kw
4. (pre-diabetes):ti,ab,kw
5. MeSH descriptor Prediabetic state explode all trees
6. MeSH descriptor Glucose Intolerance explode all trees
7. MeSH descriptor Hyperglycemia explode all trees
8. “impaired glucose regulation”:ti,ab,kw
9. “impaired glucose tolerance”:ti,ab,kw
10. “impaired fasting glycemia”:ti,ab,kw
11. (dysglyc?emia):ti,ab,kw
12. (#1 or #2 or #3 or #4 or #5 or #6 or #7 or #8 or #9 or #10 or #11)
13. (screening):ti,ab,kw
14. MeSH descriptor Mass screening explode all trees
15. MeSH descriptor Early Diagnosis explode all trees
16. “early detection”:ti,ab,kw
17. (#13 or #14 or #15 or #16)
18. (#12 AND #17)
