# Supplementary material for: Systematic Review and Meta-Analysis of Response Rates and Diagnostic Yield of Screening for Type 2 Diabetes and Those at High Risk of Diabetes
Source: PLoS One. 2015 Sep 1;10(9):e0135702. doi: 10.1371/journal.pone.0135702 (PMC4556656; doi:10.1371/journal.pone.0135702)
Supplement: S1 Sensitivity Analysis — (DOCX) [file pone.0135702.s004.docx]

**S1 sensitivity Analysis**

Table A: Estimates of the between-study variance *τ^2^* in response and yield rates on log-odds scale. 95% credible intervals (CrI) in brackets

|  | Response rate (95% CrI) % | |  | Yield rate (95% CrI) % | | |
| --- | --- | --- | --- | --- | --- | --- |
| Screening strategy | OGTT screening | Initial Invitation |  | OGTT- Diabetes | Overall- Diabetes | Diabetes + at risk of diabetes |
| One step | 1**·**10 (0**·**61, 2**·**19) | 1**·**18 (0**·**64, 2**·**46) |  | 0**·**31 (0**·**18, 0**·**58) | 0**·**52 (0**·**28, 1**·**11) | 0**·**21 (0**·**12, 0**·**40) |
| Two steps | 0**·**99 (0**·**45, 2**·**82) | 4**·**04 (1**·**51, 15**·**38) |  | 3**·**35 (1**·**42, 9**·**92) | 0**·**72 (0**·**31, 2**·**38) | 1**·**27 (0**·**41, 6**·**25) |
| Three/four steps | 0.45 (0**·**07, 6**·**04) | 1**·**10 (0**·**37, 5**·**87) |  | 2**·**49 (0**·**82, 12**·**50) | 0**·**20 (0**·**05, 1**·**28) | 2**·**06 (0**·**20, 63**·**20) |

Table B: Sensitivity analysis investigating the effect of excluding studies assessed as poor and satisfactory in quality on pooled OGTT response and yield rates. Figures are pooled mean rate with 95% credible intervals in brackets.

| Outcome | strategy | Assessment 1 | Assessment 2 | Assessment 3 |
| --- | --- | --- | --- | --- |
| OGTT Response | One Step | 62.94 (51.76, 72.86) | 65.62 (53.33, 75.82) | 65.62 (53.33, 75.82) |
| OGTT Response | Two Step | 62.55 (44.55, 78.00) | 65.16 (43.31, 82.45) | 65.16 (43.31, 82.45) |
| OGTT Response | Three/Four Step | 85.58 (77.27, 93.10) | 86.61 (75.73, 94.80) | 86.61 (75.73, 94.80) |
| OGTT Yield | One Step | 6.31 (5.07, 7.80) | 6.20 (4.88, 7.84) | 5.96 (4.63, 7.64) |
| OGTT Yield | Two Step | 13.93 (5.69, 30.95) | 13.31 (4.55, 32.48) | 13.20 (4.58, 32.30) |
| OGTT Yield | Three/Four Step | 28.27 (11.29, 54.66) | 21.44 (8.63, 45.08) | 21.36 (8.59, 44.54) |
| Assessment 1 excluded studies assessed as poor in quality  Assessment 2 excluded studies assessed as satisfactory in quality  Assessment 3 excluded studies assessed as poor and satisfactory in quality | | | | |
