# Supplementary material for: Systematic Review and Meta-Analysis of Response Rates and Diagnostic Yield of Screening for Type 2 Diabetes and Those at High Risk of Diabetes
Source: PLoS One. 2015 Sep 1;10(9):e0135702. doi: 10.1371/journal.pone.0135702 (PMC4556656; doi:10.1371/journal.pone.0135702)
Supplement: S1 WinBUGS code — (DOCX) [file pone.0135702.s005.docx]

**Supporting Information**

**S1 WinBUGS code: Random affects meta-analysis**

model{

for(i in 1:40){

tmp1[i]<-id[i] #temp variable for study id, not used

r[i] ~ dbin(p[i], n[i]) #binomial likelihood

logit(p[i]) <-delta[i] #log-odds of response

#re meta-analysis model (logit scale)

delta[i] ~ dnorm(d[steps[i]], tau[steps[i]])

}

for(j in 1:3){

d[j] ~ dnorm(0,0.00001) #Prior distribution for pooled effects

tau[j] <- pow(sd[j],-2) #between-study variance

sd[j] ~ dunif(0,2) #prior for between-study sd

res.rate[j]<- 100*exp(d[j])/(1.0+exp(d[j])) #rates (%)

NNI[j] <- 100/res.rate[j] #no. needed to invite

}

**} #End**

**Initial Values**

list(d= c(0,0,0), sd=c(1,1,1))

**Outcome data for OGGT response rate**

| # n= no. invited  # r = no. response  # steps = no of steps  #id = study identifier  #steps (1=one-step, 2=two-step,3=three/four-step strategy) | | | |
| --- | --- | --- | --- |
| id[] | n[] | r[] | steps[] |
| 1 | 540 | 449 | 1 |
| 2 | 6300 | 4733 | 1 |
| 3 | 1626 | 1034 | 1 |
| 5 | 2527 | 1522 | 1 |
| 6 | 20293 | 11247 | 1 |
| 7 | 12934 | 6784 | 1 |
| 10 | 2372 | 1317 | 1 |
| 11 | 4856 | 2595 | 1 |
| 12 | 2082 | 1374 | 1 |
| 13 | 3069 | 986 | 1 |
| 14 | 2600 | 2350 | 1 |
| 16 | 1056 | 879 | 1 |
| 17 | 2434 | 1155 | 1 |
| 18 | 4500 | 2893 | 1 |
| 19 | 5309 | 3225 | 1 |
| 20 | 1434 | 1117 | 1 |
| 21 | 11403 | 3241 | 1 |
| 23 | 5167 | 4826 | 1 |
| 25 | 30950 | 6749 | 1 |
| 26 | 22772 | 9375 | 1 |
| 29 | 2368 | 1972 | 1 |
| 30 | 1375 | 584 | 1 |
| 33 | 37 | 14 | 2 |
| 34 | 786 | 528 | 2 |
| 35 | 469 | 408 | 2 |
| 36 | 12034 | 8108 | 2 |
| 37 | 1756 | 1469 | 2 |
| 38 | 350 | 220 | 2 |
| 39 | 5000 | 3981 | 2 |
| 40 | 446 | 222 | 2 |
| 41 | 11125 | 7660 | 2 |
| 42 | 21741 | 4282 | 2 |
| 43 | 861 | 453 | 2 |
| 44 | 532 | 473 | 3 |
| 45 | 519 | 457 | 3 |
| 46 | 489 | 397 | 3 |
| 47 | 867 | 718 | 3 |
| 48 | 747 | 567 | 3 |
| 49 | 37 | 37 | 3 |
| 50 | 1687 | 1389 | 3 |
| END |  |  |  |
